# Investigation of Geographic and Macrolevel Variations in LGBTQ Patient Experiences: Longitudinal Social Media Analysis

**DOI:** 10.2196/17087

**Published:** 2020-07-31

**Authors:** Yulin Hswen, Amanda Zhang, Kara C Sewalk, Gaurav Tuli, John S Brownstein, Jared B Hawkins

**Affiliations:** 1 Bakar Computational Health Sciences Institute Department of Epidemiology and Biostatistics University of California San Francisco San Francisco, CA United States; 2 Computational Epidemiology Lab Harvard Medical School Boston, MA United States; 3 Innovation Program Boston Children's Hospital Boston, MA United States; 4 Pritzker School of Medicine The University of Chicago Chicago, IL United States

**Keywords:** LGBTQ, sexual and gender minorities, health care quality, health care disparities, social media, digital health, sentiment analysis, infodemiology

## Abstract

**Background:**

Discrimination in the health care system contributes to worse health outcomes among lesbian, gay, bisexual, transgender, and queer (LGBTQ) patients.

**Objective:**

The aim of this study is to examine disparities in patient experience among LGBTQ persons using social media data.

**Methods:**

We collected patient experience data from Twitter from February 2013 to February 2017 in the United States. We compared the sentiment of patient experience tweets between Twitter users who self-identified as LGBTQ and non-LGBTQ. The effect of state-level partisan identity on patient experience sentiment and differences between LGBTQ users and non-LGBTQ users were analyzed.

**Results:**

We observed lower (more negative) patient experience sentiment among 13,689 LGBTQ users compared to 1,362,395 non-LGBTQ users. Increasing state-level liberal political identification was associated with higher patient experience sentiment among all users but had stronger effects for LGBTQ users.

**Conclusions:**

Our findings highlight that social media data can yield insights about patient experience for LGBTQ persons and suggest that a state-level sociopolitical environment influences patient experience for this group. Efforts are needed to reduce disparities in patient care for LGBTQ persons while taking into context the effect of the political climate on these inequities.

## Introduction

### Health Disparities and Discrimination in Health Care Among Lesbian, Gay, Bisexual, Transgender, and Queer Patients

Across several health indicators, lesbian, gay, bisexual, transgender, and queer (LGBTQ) people consistently experience worse health outcomes than their non-LGBTQ counterparts [1]. Discrimination in the health care system is associated with worse health outcomes [2-4]. Previous studies have shown that LGBTQ persons report increased rates of discrimination from medical providers and other staff across a wide variety of health care settings [5,6]. In one study, almost one-third of transgender survey respondents reported postponing medical care because they experienced discrimination [6]. In addition, many LGBTQ persons report reluctance to disclose their sexual orientation or gender identity to their health care providers [7,8]. Such an environment results in a lack of understanding and acceptance of LGBTQ people and their specific health care needs, and leads to inadequate treatment and an erosion of trust in the health care services for this group [7,8]. Although there has been increasing awareness and understanding of LGBTQ patient experiences, many recent studies have consisted of small qualitative inquiries focused on specific LGBTQ populations [9-12]. Furthermore, there is a dearth of valid scales and indices that measure LGBTQ patient experience [13] as well as limited research on the sociopolitical cultural factors that contribute to these discriminatory accounts.

### Geographic Disparities in LGBTQ Health Care

There is a large body of evidence characterizing regional variation in health care and patient experiences across the United States [14-16]. Some studies have further stratified this variation by social variables such as socioeconomic status and race [15,17]. However, research has yet to explore the geographic disparities for LGBTQ care due to methodological limitations in the identification of LGBTQ patients [18]. US states vary considerably in their policies and practices that provide protection against sexual orientation and identity-based discrimination. In the areas of employment, housing, public accommodations, and health care services, there is wide variation of legislatures and pre-empt local protections that help protect LGBTQ persons by ensuring fair and equal treatment [19,20]. Although certain states have adopted protection laws for LGBTQ persons, other states have not been committed to the passage or enforcement of local nondiscrimination laws [21]. In fact, some states have policies that explicitly prevent the passage or enforcement of local nondiscriminatory laws, including those that relate to health care [21]. States without LGBTQ protective policies also tend to have higher percentages of conservative voters [22], and the social climate of a geographic area has been shown to be associated with differences in health outcomes for LGBTQ persons [23,24]. Prior research has also shown that residence in areas with a higher percentage of Republican voters is associated with a greater risk for depression among LGBTQ youth at the neighborhood level [25] and health care refusal among transgender patients at the state level [21]. In addition, physician political identity has been shown to be highly correlated with treatment decisions, with physician partisan bias leading to variation in patient care [26]. However, a large-scale, geographically contextual analysis has not been conducted to study the effect of the political environment on LGBTQ patient experience.

### Twitter as an Outlet for Patient Feedback

Recently there has been a shift in US health care to emphasize patient experiences, as this has been linked with quality of care [16]. Novel methods based on online data have been applied to studying this field [27]. For instance, the social media platform Twitter has been shown to be an effective resource for obtaining unsolicited feedback on quality of hospital care [16,27-29]. It has also been validated as a method to characterize differences in LGBTQ hospital care across the United States [30]. This previous study assessed how hospitals either supported or did not support LGBTQ care and showed that hospitals deemed as having LGBTQ equitable policies were also shown to be more supportive toward LGBTQ practices on Twitter. However, it did not investigate LGBTQ patient users’ experiences or how geopolitical state-level factors may influence the sentiment of these experiences. Other studies have demonstrated the feasibility and promise of real time social media sites to study the patient experiences of LGBTQ communities but only in small-scale content analyses, and they did not consider how geographic variation in political identity may shape these experiences [31,32]. In these ways, online social media information from LGBTQ patients can provide researchers with unfiltered accounts of patient experience and help develop an understanding of the complexities surrounding LGBTQ health disparities [33,34].

This study sought to fill the gap in research by examining the geographic variation in patient experiences among LGBTQ persons in the United States using novel computer science methods to curate a large-scale data set from the online social media platform Twitter. The goal of this research was to identify differences in patient experience among LGBTQ and non-LGBTQ patients, and to statistically model the state-level geopolitical factors that are associated with LGBTQ patient experiences using Twitter. Understanding the political factors associated with LGBTQ patient experience can be used to uncover reasons for these LGBTQ disparities in care and inform the development of targeted interventions to improve equity and advocate for this marginalized group.

## Methods

### Sample

#### Health Care Patient Experience Twitter Data

Tweets relating to patient experience from February 16, 2013, to February 15, 2017, were collected. A supervised machine classifier was built from a combination of keywords and rule-based learning algorithms to identify tweets related to health care patient experiences in the areas of medical facility and staff, medical procedures, hospital visits and stays, medications, hospital bills and insurance, care condition, and pain. Tweets repeated more than 5 times from the same user were deemed irrelevant based on manual inspection and the tweet was removed from the data set. Geolocation was collected through the metadata of Twitter. Using the geolocation inference engine, we identified the latitude and longitude of tweets. We verified the accuracy of our geolocation classifier by using Amazon Mechanical Turk (MTurk) to manually curate 10,000 randomly selected tweets and found that using MTurk validated 91% (n=9100/10,000) of the inferred locations through the geolocation engine were correct (with 87%, n=8700/10,000 agreement between two MTurk curators). Only tweets with geolocation data were used for locational analysis. The detailed methods and validation of the patient experience data set procedure is documented in Multimedia Appendix 1, which has been validated in a previous study [35].

#### LGBTQ Users

The Twitter user descriptions were collected. A user who used any of the terms “lesbian,” “gay,” “bisexual,” “transgender,” “trans,” “queer,” “LGBT,” “LGBTQ,” “intersex,” “homosexual,” or “cis” in their profile description was deemed an LGBTQ user. A non-LGBTQ user was defined as any Twitter user who did not use these terms in their profile description. The control population was comprised of the user population that did not self-identify as being LGBTQ on Twitter. The LGBTQ user was defined as a binary variable: tweets from LGBTQ users were labeled with 1, and tweets from non-LGBTQ users were labeled as 0. Tweets were labeled according to the user description at the time of the tweet; changes in LGBTQ or non-LGBTQ status over time were not considered. A conservative manual inspection of 200 user profile descriptions categorized as LGBTQ agreed 81% (n=162) of the time.

#### State Identification

Tweet latitude and longitude information were matched with the United States Census Bureau’s American Community Survey 5-year estimates to identify the corresponding state and generate a state-level field [36].

#### Geographic Regions

The geographic regions Northeast, Midwest, South, and West were determined by the US Census and were matched to the latitude and longitude of user’s tweets. The region of the Northeast includes Maine, New Hampshire, Vermont, Massachusetts, Rhode Island, Connecticut, New York, New Jersey, and Pennsylvania. The Midwest includes Ohio, Michigan, Indiana, Wisconsin, Illinois, Minnesota, Iowa, Missouri, North Dakota, South Dakota, Nebraska, and Kansas. The South includes Delaware, Maryland, Virginia, West Virginia, Kentucky, North Carolina, South Carolina, Tennessee, Georgia, Florida, Alabama, Mississippi, Arkansas, Louisiana, Texas, Oklahoma, and Washington, DC. The West comprises of Montana, Idaho, Wyoming, Colorado, New Mexico, Arizona, Utah, Nevada, California, Oregon, Washington, Alaska, and Hawaii. Graphical map descriptions of patient experience sentiment maps of LGBTQ and non-LGBTQ users were conducted using the plotly Python (Python Software Foundation) visualization library [37].

#### Political Composition

Political party affiliation at the state level was retrieved through the Gallup Daily Tracking data set [22]. Political composition was defined as percent Republican and percent Democratic for each state. Democratic advantage was defined as percent Democratic minus percent Republican by each state and was included in the analysis as a continuous variable [22]. We chose to use political affiliation data from 2015 to represent the middle point of the study time frame. There may have been changes in political affiliation, but this was not measured for this study.

#### Dependent Variable: Sentiment of Patient Experience Tweet

Sentiment analysis has been frequently used to determine the attitude and emotion of the user (ie, author) with respect to a topic [38,39]. For instance, a user might tweet “the doctors were so knowledgeable and kind. Thank you!” that would be deemed as positive, whereas a tweet from a user that states “how could you allow patients to be treated so horribly” would be determined to possess negative sentiment. The sentiment of a patient experience tweet was defined as the attitude of the patient toward their health care experience. Sentiment analysis determines the attitude of the user by measuring the polarity of the sentiment which lies in the range of -1 to 1, where 1 is an extremely positive attitude and -1 means an extremely negative attitude. We measured the sentiment polarity using the widely accepted and used lexicon and rule-based sentiment classifier called Valence Aware Dictionary for Sentiment Reasoning (VADER) to identify the sentiment of the patient experience tweet [40]. VADER computes sentiment for each word and generates compound scores for the sentence by summing the sentiment score of each word. For VADER, a sentiment score is positive if the mean compound score is greater than or equal to 0.5 and negative if the score is less than or equal to -0.5. Mean compound scores between -0.5 and 0.5 are considered neutral. Scores of exactly 0.0 are discarded as they indicate that there is not sufficient context. We expanded on VADER’s dictionary and rules to better represent the microblogging style of platforms like Twitter. This included the incorporation of *emojis* and their respective sentiment scores.

### Analysis

Data analysis was conducted using the scikit-learn [41] and statsmodels [42] packages in Python. Descriptive statistics were conducted to compare the frequency of tweets by each state-level factor between LGBTQ and non-LGBTQ Twitter users. Tweets were separated into LGBTQ user and non-LGBTQ user tweets, and the geolocation was identified. The proportion of LGBTQ or non-LGBTQ users in a region was calculated by dividing the number of either of these users in that region by the total number of users in the region. The mean democratic advantage was calculated by summing the democratic advantage of each user group and dividing it by the total number of users. Chi-square tests for proportions were conducted to compare the breakdown of LGBTQ users by geographic region compared to the breakdown of non-LGBTQ users by geographic region. An ordinary least squares regression was used to model the effect of state-level democratic advantage on patient experience sentiment while controlling for geographic region. The inclusion of an interaction term between democratic advantage and LGBTQ status allowed for LGBTQ status to moderate the effect of democratic advantage on patient experience sentiment.

#### Regression Model

tweet_sentiment ~ LGBTQ + democratic_advantage + LGBTQ * democratic_advantage + NE + S + W + ε

## Results

The total number of users in the patient experience data set was 1,376,084 users. Out of these users, 13,689 (1.00%) self-identified as LGBTQ, and 1,362,395 (99.00%) did not self-identify as LGBTQ. The number of LGBTQ users that had available geolocation data used in these analyses was 5545, and the number of non-LGBTQ users was 445,919. The data-cleaning process removed 171 out of 22,570 LGBTQ user tweets and 15,211 out of 1,946,795 non-LGBTQ user tweets. As shown in Table 1, the highest proportion for both LGBTQ and non-LGBTQ users came from the South, followed by the West, Midwest, and Northeast. A statistically significant lower proportion of LGBTQ users compared to non-LGBTQ users were present in the areas of the South and the Midwest. Alternatively, there were significantly higher proportions of LGBTQ users compared to non-LGBTQ users in the areas of the Northeast and the West. Mean democratic advantage was also significantly higher in areas with greater proportions of LGBTQ users relative to non-LGBTQ users.

**Table 1:** Descriptive analysis of LGBTQ (n=5545) and non-LGBTQ (n=445,919) tweets in the United States, 2013-2017.

Variable	LGBTQ^a^ users	Non-LGBTQ users	Test statistic	*P* value	
Northeast, n (%)	1053 (18.99)	75,761 (16.99)	3.78		<.001
South, n (%)	1786 (32.21)	167,635 (37.59)	–8.52		<.001
Midwest, n (%)	1166 (21.03)	98,354 (22.06)	–1.87		.06
West, n (%)	1540 (27.77)	104,169 (23.36)	7.29		<.001
Democratic advantage, mean	3.92	2.60	9.44		<.001

^a^LGBTQ: lesbian, gay, bisexual, transgender, and queer.

The multilevel regression output is presented in Table 2, which illustrates the association between LGBTQ individual-level status and state-level predictors of patient experience sentiment from February 2013 to February 2017. Users in the Northeast had on average a 0.0444 (*P*<.001) lower patient experience sentiment, and users in the South had on average a 0.0390 (*P*<.001) lower patient experience sentiment compared to users in the Midwest, controlling for individual-level LGBTQ status and the state-level political factor. Users in the West had on average a 0.0188 (*P*<.001) higher patient experience sentiment compared to that of users in the Midwest while controlling for all other variables in the model. Users that self-identified as LGBTQ had on average 0.0191 (*P*=.01) lower patient experience sentiment compared to users that did not self-identify as LGBTQ in areas with no difference in democratic or republican advantage, controlling for region- and political-level factors.

**Table 2:** Ordinary least squares regression results demonstrating association between LGBTQ status and state-level political leaning with patient experience sentiment in the United States, 2013-2017.

Variables	Coefficient	SE	*t* statistic	*P* value	95% CI
Intercept	–0.0407	0.001	–31.163	<.001	–0.043 to –0.038
Northeast	–0.0444	0.002	–20.199	<.001	–0.049 to –0.040
South	–0.0390	0.002	–23.217	<.001	–0.042 to –0.036
Midwest (reference group)	N/A^a^	N/A	N/A	N/A	N/A
West	0.0188	0.002	9.820	<.001	0.015 to 0.022
LGBTQ^b^ user	–0.0191	0.006	–3.408	.01	–0.030 to –0.008
Democratic advantage	0.0008	<0.0001	4.467	<.001	0.000 to 0.000
Democratic advantage x LGBTQ user	0.0014	0.001	2.713	.007	0.000 to 0.002

^a^Not applicable.

^b^LGBTQ: lesbian, gay, bisexual, transgender, and queer.

**Figure 1:**
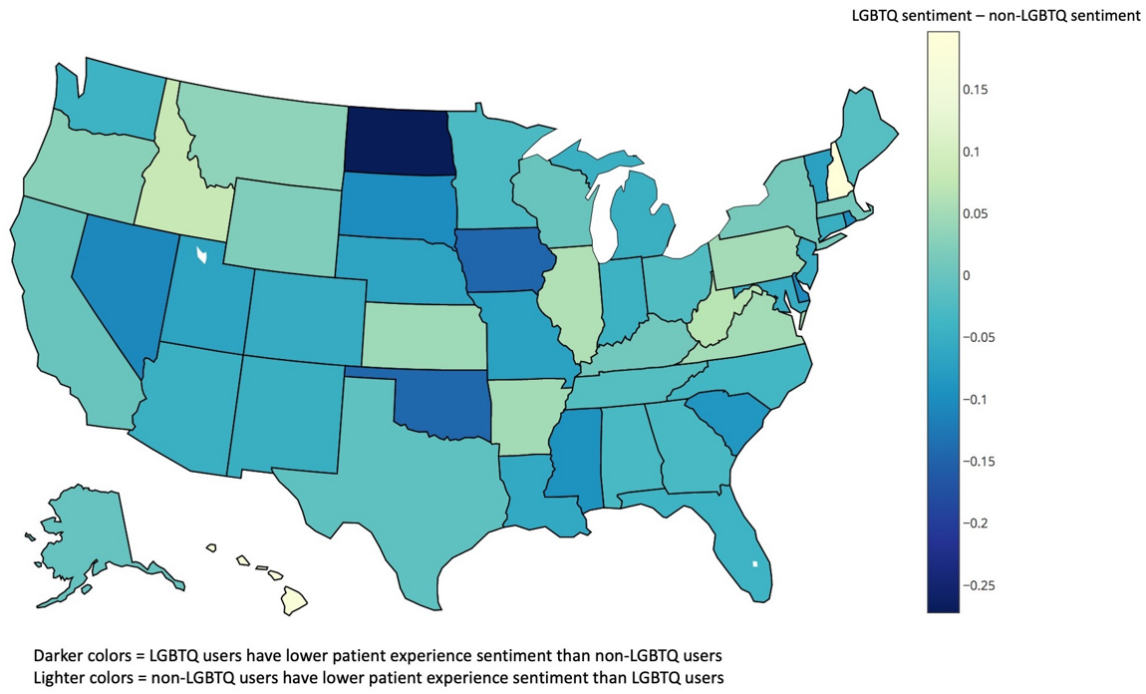
Map of patient experience tweets. LGBTQ: lesbian, gay, bisexual, transgender, and queer. (Note: Positive sentiment scores are ≥0.5; negative sentiment score ≤-0.5; neutral sentiment scores are between ±0.5).

Figure 1 shows the difference in average patient experience sentiment for LGBTQ users compared to non-LGBTQ users for each US state. Darker colors represent a more negative differential in LGBTQ user patient experience sentiment compared to non-LGBTQ users, while lighter colors represent a more positive LGBTQ user sentiment compared to non-LGBTQ users. For non-LGBTQ users, each 1-point gain in state-level democratic advantage was associated with a predicted value increase of 0.0003 in patient experience sentiment, controlling for regional variation.

The effect of democratic advantage on patient experience sentiment is moderated by LGBTQ status. Democratic advantage at the state level has a stronger effect on LGBTQ users compared to non-LGBTQ users’ patient experience sentiment. For LGBTQ users, each 1-point gain in state-level democratic advantage was associated with a predicted value increase of 0.0017, controlling for all other variables in the model. The positive effect of democratic advantage on patient experience was 5.67 times greater in LGBTQ patients than non-LGBTQ patients.

## Discussion

### Principal Findings

Our analysis highlights geographical and political factors associated with patient experience sentiment for LGBTQ persons in the United States. As documented by the online social media network Twitter, LGBTQ status was associated with worse patient experiences compared to their non-LGBTQ counterparts even after adjusting for regional variation and political stance at the state level. This is consistent with previous reports that have documented the everyday discrimination experienced by LGBTQ individuals [43]. In 2016, the Center for American Progress showed that 1 in 4 LGBTQ persons reported discrimination that compels them to make significant changes to their everyday lives to avoid that discrimination [43]. These types of changes may include health care avoidance or not sharing their LGBTQ status to medical providers or others working in the health care system. Evidence has shown strong positive associations between patient experience and clinical effectiveness for a wide range of disease areas and outcome measures [44,45]. Therefore, based on our results, poorer patient experiences among LGBTQ patients may be contributing to the disproportional burden of disease and disparity faced by LGBTQ persons [46].

Regional analysis of quality of care for the general public have indicated that areas in the South often perform more poorly on many quality measures [37]. Our results mirror such regional patterns, as we found that the South Region exhibited the worst patient experiences compared to all other regions in the United States. Findings in our study also revealed that Twitter users in the West reported the best patient experiences, which is also consistent with prior research, as this is an area that tends to perform better on the majority of quality measures [37]. However, users from the Northeast did not appear to rate their experience as well as those in the West or the Midwest. Although it has been documented that the Northeast tends to have a higher health care quality, many communities in the Northeast also have higher costs and elevated rates of avoidable hospital use, which could explain the poorer patient experience feedback reported among Twitter users in this area [36,37].

Based on our study, political values at the state level may influence the sentiment of individual-level patient experiences for LGBTQ persons more so than for non-LGBTQ persons. The political climate of a geographic area has been shown to be associated with health-related outcomes and access to care among LGBTQ persons [21,23]. However, prior studies have only focused on specific outcomes such as depression or on subsets within the LGBTQ community. For instance, one study identified that the percent of state residents voting republican was the strongest and most significant state-level predictor of health care refusal among transgender individuals [21]. Our results showed that an increasing democratic advantage at the state level contributed to better patient experiences reported at the individual level for all users even after controlling for regional variation. Furthermore, this state-level political factor had a 5.67 times stronger positive effect on patient experience sentiment for LGBTQ users compared to non-LGBTQ users. These macrolevel political affiliations based on voting behavior can potentially serve as proxies for local attitudes and culture toward LGBTQ persons, and influence structural stigmatization in health care [21,47]. It has been demonstrated that Republican-identified voters are more likely to hold discriminatory attitudes toward LGBTQ persons compared to Democratic voters [47]. Therefore, LGBTQ persons living in states with more republican voters may be more likely to encounter and receive poorer quality care from biased providers or health care systems.

### Limitations

There are limitations in this study that should be noted when interpreting our findings. First, our results examined state-level factors that were associated with patient experience sentiment and cannot be interpreted as causal. Second, the keywords methodology used to identify LGBTQ users may have incorrectly placed some LGBTQ users in the non-LGBTQ group and vice versa. The keywords list is not comprehensive and may have missed some LGBTQ-identifying descriptors such as shorthand phrases. Although we were able to manually confirm that 80-90% of LGBTQ categorized users were indeed LGBTQ users, we were not able to place a number on the accuracy of non-LGBTQ categorization. However, we believe that the percentage of LGBTQ users in the control group would not be more than the national percentage of 4.5% [48], which still provides a reasonable and valuable control population for this study. Third, there are limitations with the geo-tagged Twitter data set. Only 15% of online adults regularly use Twitter, with those 18-29 years of age being most represented. Only approximately 1% of users geo-tag the majority of the tweets they post [49,50]. Therefore, the sample population is likely not representative of the US patient population. Despite this potential limitation, many patient experience surveys have reported low response rates that range from 20-30% and have a greater proportion of older adult and female participants [51]. Our study may have a better representation of a demographic of patient participants that are normally excluded from mainstream or traditional surveys. For instance, Twitter users are generally more educated, younger, male [52], and have overrepresentation of ethnic minorities including blacks and Hispanics to a greater extent [48]. For our analysis on sentiment, previous studies have shown that the time of day, the ordering of activities within a day, and location can influence the sentiment of an individual’s tweet [49,53]. We did not control for these temporal and spatial differences, which may have influenced the results of our study.

In the past, research on LGBTQ populations has been sparse due to methodological limitations or because of issues related to homophobia (fear of people who are attracted to members of the same sex) and heterosexism (discrimination against homosexuals because of the assumption that heterosexuality is the norm of sexual orientation) [18]. Most national and state surveys lack appropriate questions pertaining to gender and sexual identity, making it difficult to conduct large-scale research [46]. Even large-scale studies typically identify few LGBTQ individuals, which has meant that LGBTQ research must rely on smaller qualitative studies or that investigation is lacking altogether [18]. Our study is the first to use a novel online social media data set captured from the popular Twitter platform to investigate LGBTQ patient experiences across the United States. Research shows that LGBTQ persons are more open to coming out and defining their LGBTQ status in their online social networks when compared to offline networks [54,55]. In fact, Twitter is a highly popular social media space for LGBTQ users when compared to other social media networks such as Facebook [55]. Finally, LGBTQ persons are more likely to search for health and medical information online when compared to their non-LGBTQ counterparts (81% vs 46%) [56]. Since LGBTQ persons face significant discrimination in typical health care settings [8], LGBTQ persons are less likely to voluntarily identify themselves as LGBTQ in the health care setting or in response to health surveys. This lack of identification prevents researchers from effectively capturing LGBTQ patient experiences. However, through our online big data set, we were able to identify an extensive number of self-identified LGBTQ users on Twitter. Based on this evidence, even though not all LGBTQ persons may have identified themselves as LGBTQ on Twitter, our study was able to capture novel insights into LGBTQ patient experiences that may not have been documented in the past.

### Conclusion

The Institute of Medicine recognizes that LGBTQ persons experience a disproportionate burden of disease and poorer health outcomes compared to the general population, and there is growing recognition of the need to further study these disparities to identify the factors that contribute to them [57]. This is the first study to leverage an online social media data set to characterize the patient experiences of LGBTQ persons. This study demonstrated that LGBTQ users experience worse patient experiences compared to non-LGBTQ users and that the political climate of a state determined through voting percentages is a prominent factor influencing patient experiences, especially for LGBTQ persons. This is especially relevant given recent emphasis on how different US government administrations can directly impact policy decisions regarding equitable health care for LGBTQ persons [58]. By identifying the factors that impact patient experience, researchers, health care providers, and policy makers can begin to develop targeted practices and policies that improve health equity for LGBTQ persons and other marginalized groups.

## Appendix

Multimedia Appendix 1 Lesbian, gay, bisexual, transgender, and queer patient experiences.

## Abbreviations

LGBTQ: lesbian, gay, bisexual, transgender, and queer
MTurk: Amazon Mechanical Turk
VADER: Valence Aware Dictionary for Sentiment Reasoning
