# Supplementary material for: Investigation of Geographic and Macrolevel Variations in LGBTQ Patient Experiences: Longitudinal Social Media Analysis
Source: J Med Internet Res. 2020 Jul 31;22(7):e17087. doi: 10.2196/17087 (PMC7428906; doi:10.2196/17087)
Supplement: Multimedia Appendix 1 [file jmir_v22i7e17087_app1.docx]

**Appendix: Patient Experience Dataset Curation**

*Extraction of tweets from Twitter*

A total of 27,309,724 tweets from February 16, 2013 to February 15, 2017 were extracted using a paid GNIP license. These tweets were queried using keyword classes and rules. The list of keyword classes and example rules are shown in Table S1. Keyword classes represent different patient experience subjects but may contain common keywords.

A rule includes multiple keyword classes appended using logical operators. For example, a rule for extracting patient experience data related to hospital staff providing hospital care would include keywords from Hospital Staff and Care Condition classes. Similarly, a rule to extract hospital bills and insurance related patient experience data would look like “Hospital Bills AND (Medical Facility OR Hospital Staff OR Medicine”. We used a total of 15 rules to query the Twitter data.

**Table S1.** Keyword classes, keywords, and example rules that were used to extract the patient experience Twitter data.

| **Keyword Classes** | |
| --- | --- |
| **Class** | **Keyword** |
| Medical Facility | Hospital, clinic, urgent care, emergency room, ED |
| Hospital Staff | Nurse, doctor, medical professional, registered nurse, dr, patient rep |
| Treatment | Treatment, treat, assist, care |
| Common Procedures | Surgery, IV, blood |
| Emergency Care | ICU, NICU, urgent care, emergency room, triage, ED |
| Hospital Bills | bill, doctor bill, health insurance |
| Care Condition | Monitor, heal, recover, care, cure, dying, dead, sicker, sick, ill, illness, condition |
| Hospital Visit | Hospital stay, hospital visit, emergency room visit |
| Pain | Pain |
| Medicine | Medicine |
| Patient Experience | Hospital experience, patient experience |
| Other | Health, patient, trauma, surgery, critical, procedure, symptoms |
| **Example Rules** | |
| 1. Medical Facility AND Hospital Staff  2. Hospital Staff AND Care Condition  3. Hospital Visit AND Hospital Staff  4. Hospital Bills AND (Medical Facility OR Hospital Staff OR Medicine)  5. Medical Facility AND Common Procedures | |

*Identification of relevant tweets using supervised machine learning classifier*

The next step was to capture the subset of tweets relevant to the patient experience. A relevant tweet included discussions about care received in a hospital, urgent care, or any other health institution—either by the person themselves, a friend, or a relative.

This task was accomplished through a supervised machine learning classifier. The classifier learned from a training dataset in which tweets were labeled as relevant or irrelevant by Amazon Mechanical Turk (MTurk) workers. In total, the MTurk curation gave us 3708 relevant and 9810 irrelevant patient experience tweets for which at least two of the MTurk curators were in agreement. A few examples of manually curated tweets are shown in Table S2. The tweets provided in this table are fictitious examples to preserve user identity and privacy.

**Table S2.** Example tweets for patient experience dataset curation.

| **Tweet Class** | **Example** |
| --- | --- |
| Relevant to patient experience | After having a tumor removed from my bladder I returned to the ward with a catheter fitted. #cityhospital |
| Irrelevant to patient experience | Need tips for better communication with your doctor? #medicine #wellness |

A variety of textual features were used for the classification task. Each tweet was tokenized using the Natural Language Toolkit TweetTokenizer.^1^ Stop words and mentions (ie words beginning with “@”) were removed. Features used included unigrams and bigrams with term frequency-inverse document frequency weights and whether the tweet contained a reference to a hospital staff member or a reference to themselves or a family member. A total of 15,000 features were used in the final model.

After evaluating a set of classifiers, we selected a support vector machine classifier that produced the highest F1 score with the lowest overfitting. The selected classifier achieved an accuracy of 83% with a precision and recall of 70% and 69%, respectively, for the patient experience tweet class. We also verified the classifier-labeled patient experience tweets by manually curating a random set of 5000 tweets and found it to be 76% in agreement with the classifier. Of the 27,309,724 tweets collected, the classifier labeled 2,759,257 tweets as relevant to patient experience.

*Geolocation of Tweets*

Twitter data rarely contains user-inputted location information. Therefore, we developed a location inference engine to approximately identify geographical locations, such as country, state, and region of the relevant tweets in this dataset.

This engine performs a set of NLP operations to parse locational strings in tweet text, remove irrelevant locations (e.g. “in your heart,” “with aliens,” “under your bed,” etc.), and input these location strings into the Google Map application programming interface.^2^ We chose to use Google’s geocoding service because it has been reported to have better accuracy,^3^ thorough coverage,^4^ and the ability to handle ambiguous location.^5^

A summary flowchart of tweet extraction, curation, and geocoding is provided in Figure S1.

**Figure S1**. Summary of patient experience dataset curation.


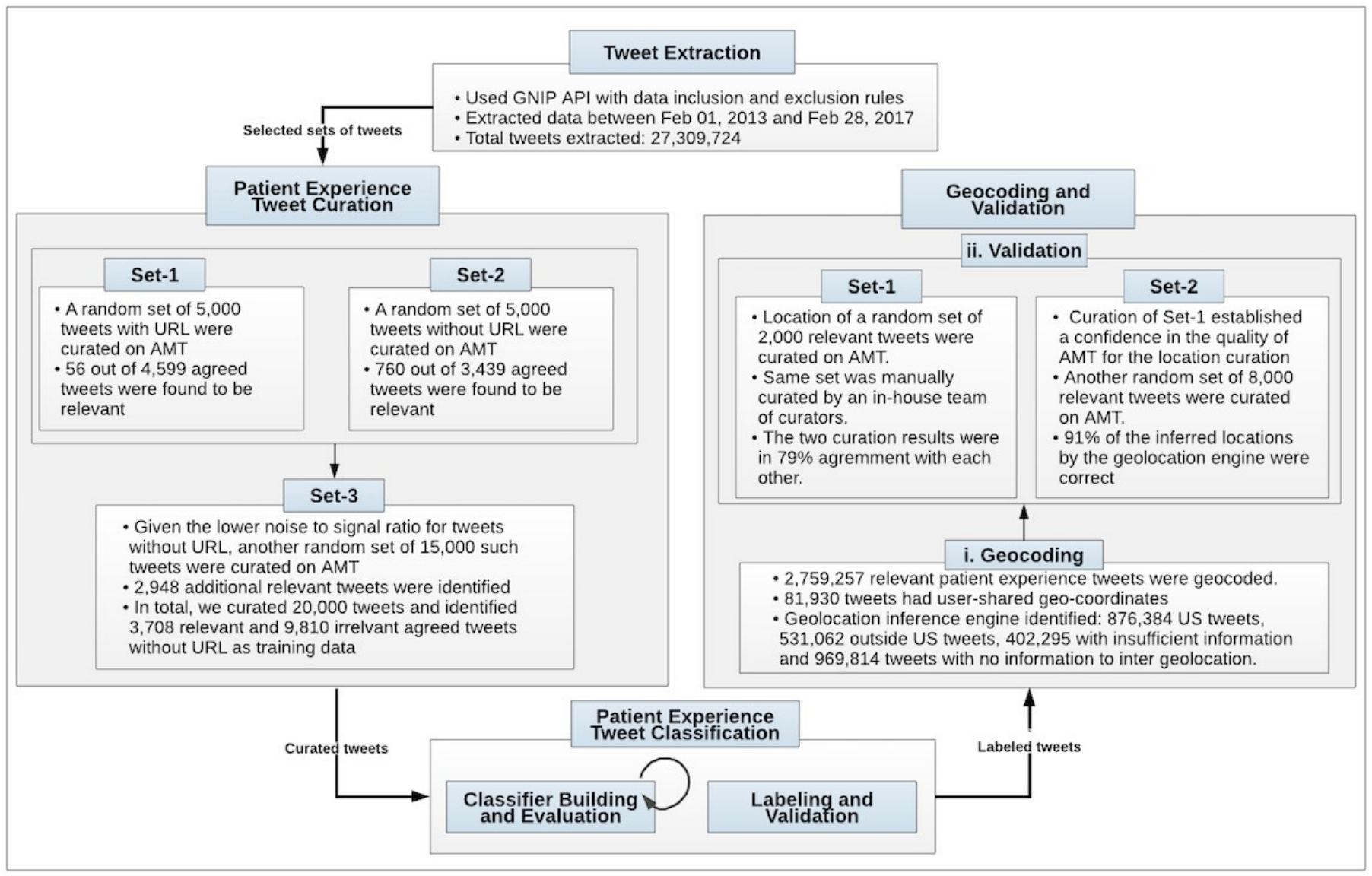


**References**

1. Loper E, Bird S. NLTK: the natural language toolkit. *arXiv preprint cs/0205028.* 2002.

2. Glickman SW, Boulding W, Manary M, et al. Patient satisfaction and its relationship with clinical quality and inpatient mortality in acute myocardial infarction. *Circulation: Cardiovascular Quality and Outcomes.* 2010;3(2):188-195.

3. Di Pietro G, Rinnone F. Online Geocoding Services: A Benchmarking Analysis to Some European Cities. Paper presented at: 2017 Baltic Geodetic Congress (BGC Geomatics)2017.

4. Ahlers D, Boll S. On the accuracy of online geocoders. *Geoinformatik 2009.* 2009.

5. Marra A. Out Online: The Experiences of LGBT Youth on the Internet. <https://www.glsen.org/press/study-finds-lgbt-youth-face-greater-harassment-online>. Accessed May 20, 2018.
